# Investigation of Absorbable and Non-Absorbable Multifilament Suture Materials in Terms of Strength Changes Using Chlorhexidine Mouthwash and Thermal Cycling: An In Vitro Study

**DOI:** 10.3390/ma17153862

**Published:** 2024-08-04

**Authors:** Ahmet Aktı, Ziya Ozan Cengiz, Gökhan Gürses, Hakan Serin

**Affiliations:** 1 Oral and Maxillofacial Department, Faculty of Dentistry, Selcuk University, Konya 42250, Turkey; dt.ahmetakti@selcuk.edu.tr (A.A.); gokhan.gurses@selcuk.edu.tr (G.G.); 2 Department of Biostatistics, Selcuk University of Veterinary Medicine, Konya 42250, Turkey; 20315901003@lisansustu.selcuk.edu.tr

**Keywords:** mouthwash, suture material, tensile strength, oral surgery

## Abstract

Sutures are natural or synthetic biomaterials utilized to hold tissues together. Following oral surgery, the surgical site and sutures are physically affected by many different factors. This study was conducted to evaluate the effect of artificial saliva (AS) and chlorhexidine mouthwash on the tensile strength of absorbable multifilament PGLA (polyglycolide-co-l-lactide) and non-absorbable multifilament silk sutures. PGLA and silk sutures, which are commonly used in oral surgery, were used to evaluate the change in strength of the sutures. A total of 352 suture samples were divided into eight equal groups (*n* = 44) and used for the experiments. Tensile strength was tested on days 0, 3, 7, and 14. For the silk sutures, there was a significant decrease in tensile strength in all groups at time T3 compared to T0, T1, and T2, and at times T1 and T2 compared to T0. For PGLA sutures, there was a significant decrease in all groups at time T3 compared to T0, T1, and T2. This study shows that chlorhexidine mouthwash significantly reduces suture resistance for 14 days after surgery.

## 1. Introduction

A suture is one of the most frequently used materials for the closure of wounds, playing a crucial role in maintaining the structural integrity of tissues. By allowing the edges of the flap to converge for a specified period, it is possible to ensure that they remain stable and facilitate an appropriate level of healing [1]. It is of paramount importance that the constant proximity of the flap edges is maintained over time, as this is a fundamental aspect of efficient wound closure. This facilitates extensive tissue healing, thereby ensuring the success of the surgical treatment [2]. Incomplete wound closure can result in wound dehiscence or prolonged healing, and failure to close the wound properly can increase the risk of infection and lead to functional problems such as pain, impaired tissue function, and chronic wounds. It may also result in the loss of soft or hard tissue grafts or implants and can lead to aesthetic problems such as changes in skin color, prominent scars, keloid formation, and tissue deformation [3,4].

One of the most crucial attributes of a suture is its robust tensile strength, which enables the surgical flaps to remain in position until the sutures are removed [5]. It is, therefore, of the utmost importance to maintain approximate wound edges with sutures that exhibit an acceptable level of tensile strength, while also ensuring minimal tissue reaction. The selection of suture material should focus on physical and biomechanical properties that will contribute to enhanced wound healing [6].

It is of great importance to be well versed in the technical properties of suture materials in order to perform suturing procedures correctly and effectively in oral surgery. A review of the literature revealed that various suture materials used in oral surgery have been evaluated [7,8]. The behavior of these materials differs from that of sutures placed in other parts of the body due to various factors, including chewing, speaking, facial movements, the presence of bacterial proteolytic enzymes, the constant presence of saliva, high tissue microcirculation, and changing pH [9]. An appropriate suture material should be capable of safeguarding the surgical field to facilitate optimal healing while maintaining minimal or no tension along the suture lines [8,10]. Therefore, it is evident that the process of suturing within the oral cavity is distinct from that employed in other areas of the body. This is because the oral cavity is a continuously functioning environment, as well as due to the presence of saliva. In summary, it is of paramount importance for dentists to possess a comprehensive understanding of the physical, mechanical, and biological properties of the suture materials that they commonly utilize [5].

The majority of studies investigating the strength of suture materials are conducted in vitro, primarily due to ethical considerations and the convenience of this approach. Nevertheless, it is challenging to recreate the oral environment through in vitro studies. The strength of the materials used is influenced by various factors, including bite forces, habits, diet, and changes in humidity and temperature [11]. The objective of cycling procedures is to modify the physical properties of a material in a manner that replicates its in vivo utilization over time. Consequently, these procedures can be employed to simulate certain clinical scenarios, thereby partially mitigating some of the limitations inherent to in vitro studies. Chlorhexidine mouthwashes are frequently employed as a means of maintaining the cleanliness of oral cavities that have been sutured. The effects of various types of mouthwash on the mechanical properties of suture material, particularly tensile strength, can be assessed by soaking sutures in appropriate solutions. This information has significant importance in guiding dentists in the selection of appropriate mouthwashes, in order to minimize the risk of suture failure and promote optimal wound healing [12]. Furthermore, a recent study has indicated that antiseptic solutions may have an impact on the failure of sutures used in knee surgery [13].

The literature has demonstrated that suture materials lose strength over time in a dry environment [14]. To mitigate this, researchers have used saline or artificial saliva (AS). However, saline solution does not have the same chemical properties as saliva, and previous studies have shown that these solutions have different effects on the strength of suture materials [15]. Research on the use of artificial saliva began in 1931, with W. Souder and W.T. Sweeney, and many researchers have aimed to develop a reference saliva over the years [16]. With technological advancements, interest in dental materials has increased, leading to the development of various types of artificial saliva. These advancements have made artificial saliva a crucial tool for in vitro studies. However, the lack of a universal model that fully replicates the biological properties of human saliva still affects the reliability of artificial saliva [16].

There are various studies in the literature focused on the suture materials used in surgery, including strength and durability analyses that compare the tensile strength and elasticity of different suture materials [3], biocompatibility studies examining their interactions with biological tissues [17], and resorption studies evaluating the degradation rates and processes of bioresorbable sutures [18]. Additionally, clinical studies have assessed the impacts of sutures on post-operative infection rates and wound healing, while material studies have focused on the development of new materials [19,20]. These research efforts aim to enhance the performance and safety of surgical sutures and to develop more effective and biocompatible materials. To the best of our knowledge, there is currently a paucity of evidence from studies evaluating the durability of multifilament sutures with different absorption properties over time when exposed to both thermal cycling and mouthwash. The objective of this study was to examine the impact of thermal cycling and chlorhexidine mouthwash on the tensile strength of multifilament suture materials with two distinct absorption properties, which are commonly used in oral surgery. The null hypothesis of the study is that mouthwashes have no effect on suture strength.

## 2. Materials and Methods

The in vitro study described in this article was conducted between February and April 2024 in the Research Laboratory of Selçuk University Faculty of Dentistry. The tensile strengths of PGLA (polyglycolide-co-l-lactide)—frequently used as an absorbable suture material in oral surgery—and non-absorbable silk multifilament suture materials were evaluated. Artificial saliva was employed as a control for assessment of the physical properties of the suture materials, while chlorhexidine mouthwash and thermal cycling were utilized to evaluate the change in tensile strength of the sutures.

### 2.1. Preparation of Solutions

The recipe described by Alsarhan et al. [14]. was used to prepare the artificial saliva. In summary, 100 mL of each of the following solutions was prepared: 25 mM potassium phosphate (K_2_HPO_4_), 24 mM sodium phosphate (Na_2_HPO_4_), 1570 mM potassium bicarbonate (KHCO_3_), and 100 mM sodium chloride (NaCl). Subsequently, the aforementioned components were combined, followed by the addition of 6 mL of 25 mM citric acid (C_6_H_8_O_7_) and 100 mL of 15 mM calcium chloride (CaCl_2_). The solution and samples were maintained at 37 °C in an incubator prior to and throughout the course of the experiment. The pH of the artificial saliva was adjusted to 6.7. Artificial saliva was used in its fresh form after preparation. The suture materials employed were 3-0 PGLA (Pegelak, Doğsan, Trabzon, Turkey) and 3-0 silk (Silk, Doğsan, Trabzon, Turkey). The efficacy of each suture material was evaluated on days 0, 3, 7, and 14. The study encompassed a total of 352 suture materials, distributed across eight distinct groups, with 44 specimens allocated to each group (Table 1). Simple sutures were applied with five knots in each sample, with all knotted specimens having the same length. Specimens exposed to chlorhexidine mouthwash (Drogsan, Kloroben, Ankara, Turkey) were treated with the test solutions for five minutes, five times a day; then washed with saline; and placed back into artificial saliva. The pH of the mouthwash solution was measured to be 5.8. All specimens were maintained in artificial saliva until the final test.

### 2.2. Thermal Cycling Process

The thermal cycling temperature was set as 5–55°, based on the recommendations of the International Standards Organization (ISO) published in 2015 [21]. The frequency of temperature changes in the oral environment is not universally agreed upon. However, many researchers suggest that one year of clinical function corresponds to 10,000 thermal cycles. This estimate is based on the hypothesis that such cycles occur 20–50 times daily, a view that is widely accepted by many authors [8]. Therefore, we decided to use 40 cycles per day. We set the saliva immersion time to 50 s, such that temperature changes could completely pass through the plastic tube and saliva could affect the suture materials. The aim was to physically age the samples in a thermal cycling device (Dental Teknik, Thermal Cycle, Turkey).

**Figure 1:**
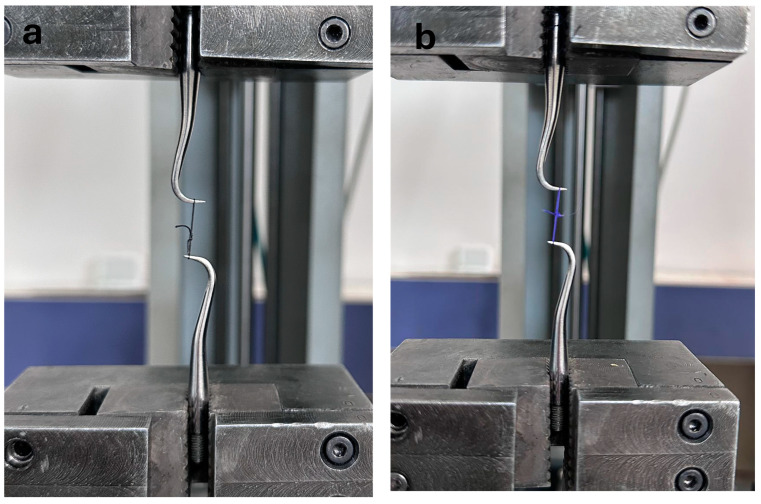
Images of the suture materials on the Instron pulling device. (**a**) Silk, (**b**) PGLA.

The mechanical properties of the suture materials were tested using a Universal Testing Machine (Instron Testing System Model 5965, Norwood, MA, USA). The tensile strength of the suture specimens was evaluated at baseline (i.e., day 0) and at days 3, 7, and 14 after immersion in the test environment. Each specimen was attached to two metal hooks located on opposite arms of the machine (Figure 1). A pilot test was performed to demonstrate that this experimental setup did not lead to failure of the hooks or knots. The samples were stretched with the help of a hook and pulled at a speed of 5 mm/min until they broke. The value at the moment of breakage was recorded as the maximum tensile strength in Newtons.

### 2.3. Statistical Analysis

All statistical analyses were conducted using the statistical software language R version 4.1.2 (The R Foundation for Statistical Computing, Vienna, Austria; https://www.r-project.org (accessed on 1 November 2021)). Before commencing the analyses, the normality of the data was evaluated using Shapiro–Wilk’s normality test and Q-Q graphs, and the assumption of sphericity was assessed with the Mauchly test. The findings of the numerical variables in the study are presented as mean ± standard deviation. A mixed ANOVA (4 repetitions: within-group comparisons and 2 factors: between-group comparisons) was conducted to assess the influences of groups and time on the tension levels of the silk and PGLA suture materials. The Bonferroni corrected *t*-test was employed for multiple comparisons.

## 3. Results

**Figure 2:**
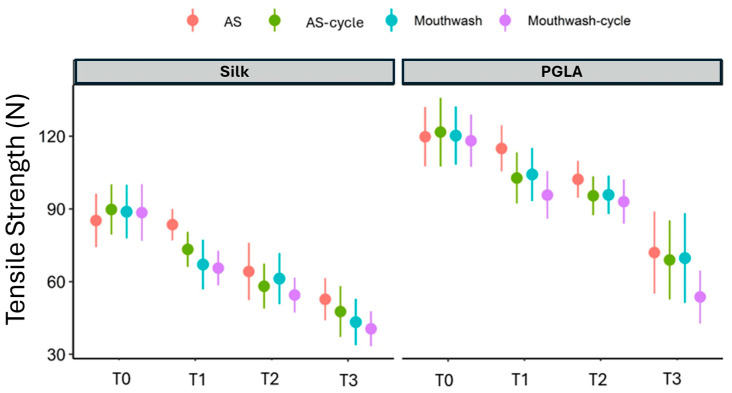
Comparison of tension levels of suture materials in AS, AS + thermal cycling, mouthwash, and mouthwash + thermal cycling treatments.

After leaving the suture materials in the test environments, all materials were found to be intact and suitable for mechanical testing. Comparisons of the tensile strength levels of the silk and PGLA suture materials at each measurement time are provided in Table 2. Graphical comparisons of the tension levels of the silk and PGLA suture materials at each measurement time are shown in Figure 2.

For the silk sutures, there was a significant decrease in salivary fluid tension levels at T2 (64.20 ± 11.78) compared to T0 (85.21 ± 11.05, adjusted *p* < 0.001) and T1 (83.51 ± 6.45, adjusted *p* < 0.001). There was also a significant decrease in tension levels at T3 (52.74 ± 8.73) compared to T0 (adjusted *p* < 0.001) and T1 (adjusted *p* < 0.001). In salivary cyclic fluid, there was a significant decrease in tension levels at T2 (58.10 ± 9.26) compared to T0 (89.79 ± 10.37, adjusted *p* < 0.001) and T1 (73.32 ± 7.23, adjusted *p* < 0.001). There was also a significant decrease in tension levels at T3 (47.65 ± 10.47) compared to T0 (adjusted *p* < 0.001) and T1 (adjusted *p* < 0.001). In mouthwash fluid, there was a significant decrease in tension levels at T3 (43.34 ± 9.55) compared to T0 (88.87 ± 11.07, adjusted *p* < 0.001), T1 (67.07 ± 10.19, adjusted *p* < 0.001), and T2 (61.24 ± 10.57, adjusted *p* < 0.001), and at T1 (adjusted *p* < 0.001) and T2 (adjusted *p* < 0.001) compared to T0. In addition, there was a significant decrease in tension levels at T3 (40.54 ± 7.18) compared to T0 (88.50 ± 11.73, adjusted *p* < 0.001), T1 (65.59 ± 7.07, adjusted *p* < 0.001) and T2 (54.46 ± 7.18, adjusted *p* = 0.039), and at T1 (adjusted *p* < 0.001) and T2 (adjusted *p* < 0.001) compared to T0.

For the Vicryl sutures, there was a significant decrease in salivary fluid tension levels at T3 (72 ± 16.93) compared to T0 (119.79 ± 12.21, adjusted *p* < 0.001), T1 (114.96 ± 9.55, adjusted *p* < 0.001), and T2 (102.21 ± 7.61, adjusted *p* < 0.001), as well as at T2 compared to T0 (adjusted *p* = 0.007) and T1 (adjusted *p* = 0.026). In salivary-circulatory fluid, there was a significant decrease in tension levels at T3 (68.94 ± 16.34) compared to T0 (121.70 ± 14.20, adjusted *p* < 0.001), T1 (102.74 ± 10.59, adjusted *p* < 0.001), and T2 (95.45 ± 7.99, adjusted *p* < 0.001), as well as at T1 (adjusted *p* < 0.001) and T2 (adjusted *p* < 0.001) compared to T0. In mouthwash fluid, there was a significant decrease in tension levels at T3 (69.72 ± 18.51) compared to T0 (120.21 ± 11.94, adjusted *p* < 0.001), T1 (104.22 ± 10.96, adjusted *p* < 0.001), and T2 (95.80 ± 7.89, adjusted *p* < 0.001), as well as at T1 (adjusted *p* = 0.001) and T2 (adjusted *p* < 0.001) compared to T0. There was a significant decrease in tension levels at T3 (53.68 ± 10.97) compared to T0 (118.14 ± 10.79, adjusted *p* < 0.001), T1 (95.73 ± 9.86, adjusted *p* < 0.001) and T2 (93 ± 9.06, adjusted *p* < 0.001), as well as at T1 (adjusted *p* < 0.001) and T2 (adjusted *p* < 0.001) compared to T0.

For the silk sutures, the tension level at T1 was significantly lower in mouthwash (adjusted *p* = 0.020) and mouthwash-cycle (adjusted *p* = 0.006) fluids compared to saliva. On the other hand, there was no difference between the tension levels of the fluids at T0, T2, and T3 (all *p* > 0.05).

For the Vicryl sutures, the tension level at T1 was significantly lower in the mouthwash/cycle fluid compared to the saliva fluid (adjusted *p* = 0.002). On the other hand, there was no difference between the tension levels of the fluids at T0, T2, and T3 (all *p* > 0.05) (Figure 2).

The overall effect of measurement times on tension levels was statistically significant (F = 492.572, *p* < 0.001, η^2^*p* = 0.860). The interaction between measurement times and suture materials was statistically significant (F = 16.008, *p* < 0.001, η^2^*p* = 0.167). The interaction between measurement times and fluids was statistically significant (F = 4.595, *p* < 0.001, η^2^*p* = 0.147). However, the effect of the interaction between measurement times, suture materials, and fluids on tension levels was not statistically significant (F = 0.905, *p* = 0.522, η^2^*p* = 0.033). The main effect of suture material on tension levels was significant (F = 316.675, *p* < 0.001, η^2^*p* = 0.798). The main effect of fluids on tension levels was significant (F = 6.557, *p* < 0.001, η^2^*p* = 0.197). On the other hand, the effect of suture–fluid interaction on tension levels was not statistically significant (F = 0.303, *p* = 0.823, η^2^*p* = 0.011).

## 4. Discussion

The unique anatomical structure of the oral cavity and its constant exposure to micro-organisms make mouth sores more susceptible to infection than those in other parts of the body. In the post-operative period, clot formation and stabilization are the basis for effective healing, and sutures play a critical role in this process. The biomechanical properties of sutures promote healing by neutralizing physiological traction forces on the edges of the healing wound [22,23]. When suture materials come into contact with saliva or other body fluids, their physical and functional properties can change, leading to the deterioration of low-tensile strength sutures during the healing process due to edema and tissue tension. Therefore, selecting sutures with high physical strength is key to successful wound healing [24]. Most sutures are made from polymers composed of repeating independent units called monomers. PGLA is one such material and has been extensively researched for applications in tissue engineering and drug delivery systems. Furthermore, various coatings are being applied to PGLA sutures to impart antibacterial properties, potentially reducing the risk of infection [25,26]. Silk is a natural material, consisting mainly of 70% protein fibers and 30% impurities or gums and, therefore, is not a polymer [12].

In this study, we compared the effects of artificial saliva and chlorhexidine-containing mouthwash on the strength of resorbable multifilament PGLA (PGLA) 3/0 and non-resorbable monofilament (silk) 3/0 sutures, which are commonly used in oral surgery, in an attempt to obtain more natural and accurate results in terms of additional factors such as thermal cycling and varying dwelling times.

A number of factors within the oral cavity can influence the strength of sutures. One such factor is the regular fluctuations in temperature that occur within the mouth. Thermal stresses can exert a pathogenic influence in two distinct ways: first, mechanical stresses caused by differential thermal changes can directly cause crack propagation through bonded interfaces. Second, altered cavity dimensions are associated with cavity volume changes that pump pathogenic oral fluids in and out of the cavities. As clinical trials are costly and time-consuming, laboratory simulations of clinical service are often performed. Therefore, we applied a thermal cycling technique to all test materials, in order to create a model as close as possible to the oral environment. Known results show that in vitro studies are generally consistent with clinical findings [27,28]. When the sutures are placed, the portion left in the tissue is resistant to proteolytic enzymes. We could not simulate this phenomenon during the thermal cycling conducted in our study.

Another important factor in studies designed to measure the strength of sutures is the calibration of the device used to apply the tensile force. The universal tester used in our study was calibrated according to the guidelines of Kim et al. [29]. In addition, the type and technique of knot tying are also important, in terms of tensile strength [30,31]. In the present study, the recommended simple suturing technique and surgeon’s knots were employed in order to prevent unraveling [14]. The selected days for the power measurements were days 3, 7, and 14. Day 3 was selected for the initial measurement of the environmental response. The rationale for selecting day 3 was that it represented the minimum time that sutures generally remain in the oral cavity [18]. Given that the optimal time for removing sutures in the oral cavity is the 14th day, our study concluded on this day [32].

The pH of the medium was adjusted according to the findings of Chu et al., given that the pH of the medium in contact with the suture material exerts a significant influence on the deterioration of the suture [32,33]. The pH of the artificial saliva was adjusted between 7.4 and 8.1 through continuous monitoring and complete replacement when a change in pH was observed, according to the findings of previous studies [8].

The TS of a suture is the maximum tension that a suture can withstand when stretched or pulled before rupture [34]. The results indicated that both sutures showed a statistically significant decrease in tensile strength from the initial tensile loads in all fluids to day 14. Previous studies have reported that resorbable materials have a higher resistance to stress, while non-resorbable materials have lower resistance. The obtained results were in agreement with Moser et al., who found that PGLA showed the best results under both natural conditions and after immersion in Ringer’s solution for 5 to 7 days [35]. However, we observed a significant decrease in the resistance of PGLA after 7 to 14 days in the thermal cycling groups. The strength values of PGLA were initially higher than those of silk and decreased to almost the same level as silk in all fluids by day 14. The dramatic decrease observed from day 7 to day 14 can be attributed to the resorption process of PGLA.

Some studies have shown that pH levels may have a greater effect on the performance of absorbable than non-absorbable sutures. Both acidic and alkaline environments can accelerate the degradation of absorbable sutures. Among non-absorbable sutures, silk appears to be the most sensitive to variation in pH conditions [33]. Silk is the most widely used material, despite its poor mechanical properties, possibly due to its ease of use, reliability, and durability. In contrast, some studies have concluded that PGLA sutures achieve the best durability under acidic pH conditions [36]. In our study, the differences observed in suture types when exposed to artificial saliva and mouthwash solutions at different pH levels suggest that ambient pH has a significant effect on the mechanical strength of sutures, as reported in the literature.

It has been observed that absorbable materials demonstrate greater stress resistance, while non-absorbable materials exhibit lower resistance [37]. The findings of Khiste et al. indicated that polyglactin (PGLA) sutures demonstrated consistent tensile strength up to day 10 but exhibited a notable decline in strength by day 14 [38]. The results of our study indicated a notable decline in the tension levels in the saliva and saliva + thermal cycling groups over time in the measurements taken for the silk suture material. The observed reductions in the physical properties of suture materials demonstrate the influence of the fluids on these materials. The observation that these decreases were more pronounced, particularly on the 7th and 14th days, indicates that the performance of the material during the healing process is influenced by the type of fluid that it is immersed in. This finding is consistent with the results of previous studies [12,39]. A study was conducted to analyze the effects in the mouthwash and mouthwash + thermal cycling groups. The results indicated that the lowest tension levels were recorded on the 14th day. This indicates that the mouthwash + cycling technique may be more effective than other methods in reducing the strength of suture materials. The PGLA results revealed that significantly lower tension levels were observed in the mouthwash + thermal cycling group on day 3, in comparison to the other groups. Nevertheless, no discernible variations were observed between the different liquid types at other times. In a separate study by Ferguson et al., it was demonstrated that saliva-soaked PGLA had a lower tensile strength than that soaked in saline or milk. To substantiate this assertion, the authors posited that saliva accelerates deterioration of the suture, thereby reducing its tensile strength [40]. In a separate in vitro study conducted by Khiste et al., PGLA sutures demonstrated a reduction in tensile strength when subjected to simulated oral conditions. These sutures exhibited consistent strength up to day 7 and day 10, with a notable decline in strength observed on day 14 [38]. The results of our study were consistent with those previously reported by Ferguson and Khiste. When silk and PGLA materials were compared, the effects of different fluids were found to be similar on day 7. However, the effect of mouthwash use on the strength of sutures was found to increase dramatically throughout the study period. Consequently, it is recommended that particular consideration be given to the use of mouthwash after the seventh day during the healing process.

However, it should be noted that the applicability of the results of this study to a clinical setting is limited due to several methodological limitations. As this was an in vitro study, despite our efforts to simulate the oral environment in terms of temperature and pH, we could not include the microbiological component and organic compounds present in natural saliva, which could significantly impact our results. Furthermore, the effects of muscle movements during oral functional activities such as chewing, swallowing, and speaking could not be fully replicated in a laboratory setting.

## 5. Conclusions

This study yielded significant insights into the impact of chlorhexidine mouthwash on the tensile forces of diverse suture materials employed in the oral environment over time. The findings indicated that chlorhexidine mouthwash may significantly reduce the resistance of suture materials for 14 days following surgery. This decrease in the tensile strength of suture materials suggests that chlorhexidine mouthwash should be used with greater caution during the post-operative healing process. In future studies, a more comprehensive investigation of the effects of chlorhexidine mouthwashes used at different concentrations and durations on suture materials is expected to further contribute to the knowledge base in this field. Additionally, further studies are needed to provide a scientific explanation for the time-dependent changes in the tensile strength of the tested materials under the given conditions. These studies will ensure that the results obtained are more comprehensive and reliable.

## Figures and Tables

**Table 1:** Conditions defining the experimental groups.

Groups	
Silk Group 1 (*n* = 44)	Silk suture soaked in artificial saliva
Silk Group 2 (*n* = 44)	Silk suture soaked in artificial saliva using thermal aging technique
Silk Group 3 (*n* = 44)	Silk suture exposed to chlorhexidine-containing mouthwash
Silk Group 4 (*n* = 44)	Silk suture exposed to chlorhexidine-containing mouthwash after thermal aging technique
Vicryl Group 1 (*n* = 44)	Vicryl suture soaked in artificial saliva
Vicryl Group 2 (*n* = 44)	Vicryl suture aged in artificial saliva using thermal aging technique
Vicryl Group 3 (*n* = 44)	Vicryl suture exposed to chlorhexidine-containing mouthwash
Vicryl Group 4 (*n* = 44)	Vicryl suture exposed to chlorhexidine-containing mouthwash after thermal aging technique

**Table 2:** Tension levels of silk and PGLA suture materials compared to fluids at each measurement time. Lowercase indicates significant difference between times in each liquid for silk and PGLA suture materials; uppercase indicates significant difference between fluids for silk and PGLA sutures at each time point.

	Tensile Strength (N)			
	T0 (Day 0)	T1 (Day 3)	T2 (Day 7)	T3 (Day 14)
Silk Group 1 (*n* = 44)	85.21 ± 11.05 ^a,A^	83.51 ± 6.45 ^a,A^	64.20 ± 11.78 ^b,A^	52.74 ± 8.73 ^b,A^
Silk Group 2 (*n* = 44)	89.79 ± 10.37 ^a,A^	73.32 ± 7.23 ^ab,B^	58.10 ± 9.26 ^c,A^	47.65 ± 10.47 ^c,A^
Silk Group 3 (*n* = 44)	88.87 ± 11.07 ^a,A^	67.07 ± 10.19 ^b,B^	61.24 ± 10.57 ^b,A^	43.34 ± 9.55 ^c,A^
Silk Group 4 (*n* = 44)	88.50 ± 11.73 ^a,A^	65.59 ± 7.07 ^b,B^	54.46 ± 7.18 ^b,A^	40.54 ± 7.18 ^c,A^
PGLA Group 1 (*n* = 44)	119.79 ± 12.21 ^a,A^	114.96 ± 9.55 ^a,A^	102.21 ± 7.61 ^b,A^	72 ± 16.93 ^c,A^
PGLA Group 2 (*n* = 44)	121.70 ± 14.20 ^a,A^	102.74 ± 10.59 ^b,AB^	95.45 ± 7.99 ^b,A^	68.94 ± 16.34 ^c,A^
PGLA Group 3 (*n* = 44)	120.21 ± 11.94 ^a,A^	104.22 ± 10.96 ^b,AB^	95.80 ± 7.89 ^b,A^	69.72 ± 18.51 ^c,A^
PGLA Group 4 (*n* = 44)	118.14 ± 10.79 ^a,A^	95.73 ± 9.86 ^b,B^	93 ± 9.06 ^b,A^	53.68 ± 10.97 ^c,A^

## Data Availability

The original contributions presented in the study are included in the article, further inquiries can be directed to the corresponding author.
